# Recent Advances in Endometrial Cancer

**DOI:** 10.12688/f1000research.10020.1

**Published:** 2017-01-27

**Authors:** Arthur-Quan Tran, Paola Gehrig

**Affiliations:** 1 Gynecologic Oncology, University of North Carolina at Chapel Hill, NC, USA

**Keywords:** endometrial cancer, surgical therapies, treatment, molecular targets

## Abstract

Endometrial cancer is the most common gynecologic malignancy in the United States, with yearly rates continuing to increase. Most women present with early stage disease; however, advanced disease carries a grave prognosis. As a result, novel therapies are currently under investigation for the treatment of endometrial cancer. These advances include a better understanding of the genetic basis surrounding the development of endometrial cancer, novel surgical therapies, and new molecular targets for the treatment of this disease. This review explores the literature regarding these advancements in endometrial cancer.

## Introduction

Endometrial carcinoma is the most common gynecologic malignancy in the United States, with approximately 60,050 newly diagnosed cases and 10,470 deaths expected in 2016. Additionally, the incidence of endometrial carcinoma is estimated to increase by 1–2% yearly ^1^. Most women are diagnosed at an early stage and have relatively good survival rates; however, women who are diagnosed with advanced-stage or recurrent disease have a poor prognosis ^2^. Thus, novel therapies are being investigated to combat the increasing disease burden of endometrial carcinoma.

Treatment modalities in endometrial cancer vary depending on the grade and the stage of the disease. Currently, the treatment and staging of endometrial carcinoma is primarily surgical, with hysterectomy and bilateral salpingo-oophorectomy being the standard of care. The issue of lymphadenectomy remains under debate, but in the U.S. it is generally performed based on criteria such as grade, depth of invasion, and tumor size. Sentinel lymph node (SLN) sampling has been advocated as an alternative to standard hysterectomy with complete lymphadenectomy. Following surgical treatment, patients may receive adjuvant radiation, chemotherapy, or both, depending on the stage and other pathologic features of their disease.

We will review the available literature regarding the current understanding of endometrial cancer, including genetic background, molecular targeting, surgical management, and adjuvant treatment.

## Genetic basis of endometrial cancer

Among adenocarcinomas of the endometrium, two distinct histologic categories have been described – type 1 and type 2 ^3^. Type 1 endometrial carcinomas account for 70–80% of new cases ^3^. These cancers are of endometrioid histology and lower grade. Type 1 cancers are estrogen mediated with high rates of K-ras and PTEN loss or mutation; there are also defects in mismatch repair (MMR) genes leading to microsatellite instability (MSI) ^4– 8^. Women with these cancers are often obese with evidence of endogenous estrogen excess. Type 2 endometrial carcinomas, on the other hand, occur in older women who traditionally were thought to be leaner, though these patients are also of increasing BMI. These cancers show aneuploidy, p53 mutations, and overexpression of HER-2/neu ^9– 15^. Type 2 endometrial carcinomas consist of higher-grade adenocarcinomas and the non-endometrioid histologies.

Though most endometrial cancer is sporadic, a significant proportion of endometrial cancers are due to inherited genetic mutations. Specifically, Lynch syndrome accounts for 2–5% of all endometrial carcinomas ^16, 17^. In women with Lynch syndrome, their risk of endometrial cancer approaches 70% ^18, 19^. Lynch syndrome is caused by germline mutation in one of many DNA MMR genes: *MLH1*, *MSH2*, *MSH6*, and *PMS2* ^20, 21^. MMR proteins are responsible for maintaining genomic integrity by correcting base substitution mismatches and insertion-deletion mismatches resulting from DNA replication errors. MMR mutations cause alterations within microsatellite regions, resulting in MSI. The MSI may lead to downstream consequences with genetic expression, resulting in aberrant cell growth or cell death ^22^.

Testing for Lynch syndrome generally begins with testing the tumor specimen. Initially, the tumor is tested using immunohistochemistry (IHC) to evaluate MMR protein levels. If the IHC suggests the possibility of an MMR gene defect, the tumor is tested for MSI through polymerase chain reaction ^23, 24^. Depending on the levels of MSI detected, detection of MMR protein loss, germline mutation testing, and specific methylation status of genetic promoter regions, patients can be found to have Lynch syndrome mutations. While there is no consensus on the sequence of IHC or MSI testing and both can be concurrently examined, our practice is to order IHC testing followed by MSI testing based on both the results of IHC and clinical suspicion.

Because of the potential lifetime risk of developing a subsequent malignancy, the Society of Gynecologic Oncology advised that all women with endometrial cancer be assessed for Lynch syndrome. Additionally, women with a family history of endometrial cancer or colon cancer should pursue genetic counseling and testing ^16^.

Data to date have been limited in associating endometrial cancer with hereditary breast and ovarian cancer syndromes. Previous literature has been conflicting on the association between *BRCA* mutations and serous endometrial carcinoma ^25, 26^. Recent evidence shows a potential link between *BRCA1* and the subsequent development of serous endometrial carcinoma ^27^. Further evidence is still needed to establish a firm genetic link. However, there should be a discussion of including prophylactic hysterectomy at the time of risk reduction bilateral salpingo-oophorectomy in *BRCA1*-affected women.

## Novel surgical treatment

Surgery remains a mainstay of treatment for most women with endometrial cancer. Since 1988, the International Federation of Gynecology and Obstetrics (FIGO) requires that staging of endometrial cancer occur surgically. Surgery includes hysterectomy with possible removal of fallopian tubes and ovaries bilaterally and consideration of lymph node assessment. There are many nuances involved in the surgical and adjuvant management of patients with endometrial cancer; both the American College of Obstetricians and Gynecologists (ACOG) and the Society of Gynecologic Oncology (SGO) have suggested that patients would benefit most from their surgery being performed by surgeons with training in gynecologic oncology ^28^.

The requirement for surgical staging reflects the increasing data on the prognostic significance of lymph node status and the implications for treatment in node-positive cancers; however, controversy exists on the role of lymphadenectomy in endometrial carcinoma. Retrospective analyses reveal a theoretical benefit to debulking clinically enlarged lymph node metastasis and a benefit to resection of microscopic metastasis in high-risk endometrial cancers ^29– 35^. Randomized trials show no therapeutic benefit to lymphadenectomy, though these trials comprise predominantly low-risk endometrial cancer histologies ^36, 37^. The risk of lymphedema and increased surgical complications form the basis for the argument against lymphadenectomy ^38^. Additional evidence is necessary in order to reach consensus regarding the benefits of lymphadenectomy compared to the risks of increased surgical procedures.

The SLN technique attempts to strike a balance between the risks and benefits of surgical lymph node evaluation. The SLN represents the first node to drain a tumor site and often is the first site of occult malignancy. If an SLN can be accurately identified and there is a high amount of certainty in detecting metastatic disease within the SLN, this technique obviates the need for a complete lymphadenectomy. Thus, the viability of this technique depends on the ability of dye or tracer to map from the tumor to the SLN. The complicating factor of the SLN technique is that the lymphatic drainage of the endometrium is complex, unlike in breast or vulvar cancer.

Because of the location of the disease, endometrial tumors are less readily accessible for peritumoral injection. Several techniques have been described for injecting dye either through the cervix, via hysteroscopy, or through fundal injections. Cervical injections have been the easiest to perform and have been found to have SLN detection rates that are comparable to other described methods ^39, 40^.

Various dyes and tracers have been used in endometrial cancer in an attempt to improve SLN detection, each with their own unique risks and benefits. Isosulfan blue is a dye that works by staining the lymph nodes and lymph vessels, and it is one of the most frequently used methods for SLN detection – the colorimetric method. Cervical injection of this dye requires no specialized equipment; however, visualization of dye in obese patients is inferior to visualization of dye in non-obese patients. Technetium sulfur colloid (Tc ^99^) is a radioactive tracer able to be detected by gamma probes. When using technetium, preoperative lymphoscintigraphy and a handheld gamma probe can be used to map lymphatic drainage. This technique also has its own limitations, including additional operative time, coordination of procedures, and evidence of poor correlation between lymphoscintigraphy and surgical SLN mapping ^41^. Lastly, indocyanine green (ICG) has been reported to have excellent signal uptake while allowing for real-time visualization of lymphatic drainage using near-infrared fluorescence imaging. Bilateral detection rates with ICG are comparable or better than either Tc ^99^ or blue dye ^42^.

The crux of the SLN technique lies within its diagnostic accuracy. In a prospective multicenter study, patients with early stage disease underwent SLN assessment with a combination of dyes followed by pelvic-node dissection. The overall predictive value of the SLN technique was found to be 97%. The patients who did not have positive lymph nodes detected had type 2 endometrial cancer ^43^. The false negative results of this trial underscore a potential limitation surrounding SLN techniques.

In high-risk (type 2) endometrial cancers, the application of the SLN technique remains controversial. Patients with high-risk endometrial cancer are at a higher risk for unsuccessful mapping and isolated positive para-aortic lymph nodes. Retrospective series have suggested similar outcomes in women with high-grade cancers undergoing the SLN technique or a complete lymphadenectomy; however, data from prospective trials remain lacking ^44, 45^.

If the SLN technique is to be used, it is important to adhere to the National Comprehensive Cancer Network (NCCN) guidelines. According to the guidelines, lymph nodes that are mapped or look suspicious should be removed. If there is no mapping on a hemi-pelvis, the NCCN guidelines suggest performing a side-specific lymphadenectomy. The necessity for a para-aortic dissection is left to the discretion of the surgeon.

## Molecular targeted therapies

Our understanding of endometrial cancer has shifted dramatically. Historically, endometrial cancer has been designated as type 1 and type 2, each type being associated with its own genetic aberrations. As we discussed previously, type 1 endometrial cancers have deletions in K-ras, PTEN, or MMR ^4– 8, 46– 48^. Conversely, type 2 endometrial cancers show aneuploidy, p53 mutations, and overexpression of Her-2/neu ^9– 15^. Using integrated genomics and epigenomic, transcriptomic, and proteomic techniques, The Cancer Genome Atlas (TCGA) has recently provided compelling evidence that endometrial cancers can be classified into four categories: polymerase epsilon (POLƐ) ultramutated, MSI hypermutated, copy-number low, and copy-number high, serous-like ^49^.

Our understanding of the genetic aberrations of endometrial cancers may represent a better tool to classify and guide future therapies towards more biologically aggressive diseases. Common targets for therapeutics involve drugs that affect apoptosis, signal transduction, epigenetic modification, cell cycle progression, protein folding and degradation, hormone receptor activation, and angiogenesis. We will be focusing on the uses of anti-angiogenic agents, epidermal growth factor receptor (EGFR) inhibitors, HER2/neu antibodies, and phosphoinositide 3-kinase (PI3K)-PTEN-AKT-mammalian target of rapamycin (mTOR) pathway inhibitors in endometrial cancer (see Table 1). Further targets, novel therapies, and genome-guided clinical trials may arise as we gain a deeper understanding of the molecular pathways and genetic aberrations in endometrial cancer.

**Table 1.** Targeted therapies in endometrial cancer.

Treatment	Molecular Target	Phase of Study	Population	Results
Bevacizumab	VEGF-A	II ^57^	Recurrent or persistent endometrial cancer	RR: 7/52 (13.5%) CR: 1/52 (2%) PR: 6/52 (11.5%)
		II ^58^	Advanced or recurrent endometrial cancer	RR: 11/15 (73%) CR: 5/15 (33%) PR: 6/15 (40%)
*with temsirolimus*		II ^59^	Recurrent or persistent endometrial cancer	RR: 12/49 (24.5%) CR: 1/49 (2%) PR: 11/49 (22%)
*with radiation therapy*		II ^60^	Recurrent endometrial or ovarian cancer	RR: N/A
*with radiation therapy*		II ^61^	Endometrial cancer with high-risk factors	RR: N/A
*with chemotherapy*		II ^62^	Advanced or recurrent endometrial cancer	RR: N/A
Thalidomide		II ^63^	Recurrent endometrial cancer	RR: 3/24 (12.5%) CR: 0/24 (0%) PR: 3/24 (12.5%) SD: 2/24 (8%)
Aflibercept	VEGF-A and VEGF-A isoforms	II ^64^	Recurrent or persistent endometrial cancer	RR: 3/42 (7%) CR: 0/42 (0%) PR: 3/42 (7%)
Sorafenib	TKI, VEGF receptors	II ^65^	Advance uterine carcinoma or carcinosarcoma	RR: 2/40 (5%) CR: 0/40 (0%) PR: 2/40 (5%) SD: 17/40 (42.5%)
Dovitinib	TKI, VEGF receptors	II ^68^	Progressive or advanced endometrial cancer	RR: 6/53 (11%) CR: 0/53 (0%) PR: 6/53 (11%)
Nintedanib	TKI, VEGF receptors	II ^67^	Recurrent or persistent endometrial cancer	RR: 3/32 (9%) CR: 0/32 (0%) PR: 3/32 (9%)
Brivanib	TKI, VEGF receptors	II ^69^	Recurrent or persistent endometrial cancer	RR: 8/43 (19%) CR: 1/43 (2%) PR: 7/43 (17%)
Sunitinib	TKI, VEGF receptors	II ^66^	Recurrent or metastatic endometrial cancer or carcinosarcoma	RR: 6/33 (18%) CR: 0/33 (0%) PR: 6/33 (18%) SD: 10/33 (30%)
Gefitinib	EGF receptors	II ^74^	Recurrent or persistent endometrial cancer	RR: 1/26 (4%) CR: 1/26 (4%) PR: 0/26 (0%) SD: 7/26 (27%)
Erlotinib	EGF receptors	II ^73^	Advanced or recurrent endometrial cancer	RR: 4/32 (12.5%) CR: 0/32 (0%) PR: 4/32 (12.5%) SD: 15/32 (47%)
Lapatinib	EGF receptors	II ^75^	Recurrent or persistent endometrial cancer	RR: 1/30 (3%) CR: 0/30 (0%) PR: 1/30 (3%) SD: 7/30 (23%)
Trastuzumab	HER2/neu	II ^79^	Advanced or recurrent endometrial cancer	RR: 0/33 (0%) CR: 0/33 (0%) PR: 0/33 (0%) SD: 12/33 (36%)
Ridaforolimus	mTOR	II ^82^	Advanced endometrial cancer	RR: 0/64 (0%) CR: 0/64 (0%) PR: 0/64 (0%) SD: 22/64 (35%)
		II ^83^	Advanced or recurrent endometrial cancer	RR: 3/31 (9%) CR: 0/64 (0%) PR: 3/31 (9%) SD: 18/34 (53%)
		II ^84^	Advanced endometrial cancer	RR: 5/45 (11%) CR: 0/45 (0%) PR: 5/45 (11%) SD: 8/45 (18%)
Everolimus	mTOR	II ^86^	Recurrent endometrial cancer	RR: 0/28 (0%) CR: 0/28 (0%) PR: 0/28 (0%) SD: 12/28 (43%)
*with letrozole*	mTOR	II ^87^	Recurrent or progressive endometrial cancer	RR: 11/35 (32%) CR: 9/35 (26%) PR: 2/35 (6%) SD: 4/35 (11%)
Temsirolimus	mTOR	II ^88^	Advanced or recurrent endometrial cancer	RR: 9/54 (17%) CR: 0/54 (0%) PR: 9/54 (17%) SD: 32/54 (59%)
*with bevacizumab*		II ^59^	Recurrent or persistent endometrial cancer	RR: 12/49 (24.5%) CR: 1/49 (2%) PR: 11/49 (22.5%)
Pilaralisib	PI3K	II ^90^	Advanced or recurrent endometrial cancer	RR: 4/67 (6%) CR: 2/67 (3%) PR: 2/67 (3%) SD: 25/67 (37%)
GDC-0980	PI3K/mTOR	II ^91^	Recurrent or persistent endometrial cancer	RR: 4/55 (7%) CR: 2/55 (3.5%) PR: 2/55 (3.5%)

CR, complete response; EGF, epidermal growth factor; HER, human epidermal growth receptor; mTOR, mammalian target of rapamycin; PI3K, phosphoinositide 3-kinase; PR, partial response; RR, response rate; SD, stable disease; TKI, tyrosine kinase inhibitor; VEGF, vascular endothelial growth factor.

### Anti-angiogenic agents

Once a tumor reaches a critical point of hypoxemia, malignancies require proliferation of new blood vessels, or angiogenesis, in order to grow, progress, and metastasize ^50– 52^. Vascular endothelial growth factor (VEGF) induces new blood vessel formation and has been associated with a poor prognosis. Specifically, in endometrial cancer, VEGF has been correlated with deep myometrial invasion, higher histologic grade, lymphovascular space invasion, and lymph node metastasis ^50, 53– 56^. VEGF is consistently expressed in a majority of endometrial cancers ^57^.

Several studies have sought to take advantage of VEGF as a target in an attempt to improve outcomes in patients with endometrial cancer; the results of these trials have had mixed results. Bevacizumab (Avastin®, Genentech) is a recombinant humanized monoclonal antibody against VEGF-A and has been shown to have the most promise in terms of clinical response rates in recurrent or advanced endometrial cancer. There is evidence of moderate response rates with slight increases in progression-free survival (PFS) in multiple phase II trials using bevacizumab either alone or in combination with an mTOR inhibitor ^58– 60^. Additionally, early studies adding bevacizumab to radiation therapy for endometrial cancer have shown improved local control ^61, 62^. An ongoing phase II trial using bevacizumab in combination with cytotoxic agents was presented at the 2015 American Society of Clinical Oncology (ASCO) Annual Meeting. Thus far, the results are promising, with a potential survival benefit (NCT00977574) ^63^. There have yet to be phase III data with regard to the use of bevacizumab for endometrial cancer.

Aside from bevacizumab, other anti-angiogenic drugs have been evaluated with limited success. Thalidomide, which has an unknown mechanism of action as an anti-angiogenic agent, has been previously explored for single-agent use in endometrial cancer; however, the response rate was not deemed sufficient to proceed with further investigation ^64^. Aflibercept, a VEGF-trap, is a human immunoglobulin G that acts as a decoy receptor to bind VEGF-A and neutralize VEGF-A isoforms. Initial work showed promise in endometrial cancer; however, a phase II trial using single-agent aflibercept showed a low response rate and significant toxicity ^65^. Sorafenib, a multiple-targeted kinase inhibitor that also inhibits VEGF receptors, has shown limited activity in endometrial cancer ^66^. Similarly, there are multiple small molecule tyrosine kinase inhibitors (TKIs) that have inhibitory activity at the VEGF receptor. These include dovitinib, nintedanib, brivanib, and sunitinib. The results from phase II trials with these agents showed limited overall activity ^67– 70^. Although results have been disappointing thus far, promising findings involving anti-angiogenic agents in other tumor sites may ultimately yield solutions in endometrial cancer.

### EGFR inhibitors

EGFR overexpression is common in endometrial cancer and has been correlated with tumor grade, deep myometrial invasion, and poor prognosis ^71– 73^. The EGFR family consists of four distinct tyrosine kinase cell-surface receptors: ErbB-1 (EGFR), ErbB-2 (HER2/neu), ErbB-3, and ErbB-4. Despite the success of EGFR inhibitors in other malignancies, discouraging results have been observed in endometrial cancer owing to low response rates to drugs. Specifically, gefitinib, erlotinib, and lapatinib – all orally available inhibitors of EGFR – did not show high levels of clinical benefit in phase II trials ^74–76^.

### HER2/neu inhibitors

The HER2/neu protein consists of a cysteine-rich extracellular ligand-binding domain, a hydrophobic membrane-spanning domain, and an intracellular tyrosine kinase domain. The overexpression of HER2/neu results in modulation of cell proliferation, differentiation, migration, and survival and upregulation of the PI3K/AKT/mTOR and Ras/Raf/MAPK pathways ^77^. Furthermore, HER-2/neu overexpression has been demonstrated in advanced endometrial cancers – specifically, type 2 cancers – and is associated with a poor prognosis ^78, 79^. Trastuzumab, a monoclonal antibody that interferes with HER2, showed limited activity in one phase II trial; the trial was ultimately closed because of poor accrual ^80^. A randomized phase II trial is currently ongoing which evaluates the use of carboplatin and paclitaxel chemotherapy in conjunction with trastuzumab (NCT01367002). The trial may clarify the utility of this monoclonal antibody on patients with HER2/neu-overexpressing endometrial cancers.

### PI3K-PTEN-AKT-mTOR pathway inhibitors

Endometrial cancer demonstrates the highest rate of PI3K pathway alterations of all solid tumors ^81^, and 40–80% of women with type 1 endometrial cancers harbor PTEN mutations ^6– 8^. PTEN acts similarly as a tumor suppressor; it inhibits cell adhesion and migration and antagonizes the PI3K/AKT/mTOR pathway. The loss of PTEN, therefore, results in the activation of AKT, which subsequently upregulates mTOR activity. Thus, mTOR inhibitors are becoming an appealing class of drug owing to their ability to modulate signal transduction pathways involved in cell cycle progression ^82^. Unfortunately, the results for trials involving inhibitors of this pathway have shown very weak response.

mTOR inhibitors evaluated in endometrial cancer include ridaforolimus, everolimus, and temsirolimus. Ridaforolimus has been investigated and compared to hormones and chemotherapy in multiple phase II trials. The results of the trials have shown weak response rates with only modest levels of stable disease and significant toxicity ^83– 85^. Phase II trials with everolimus alone showed weak results, but the results were more promising when it was used in combination with letrozole ^86– 88^. The combination showed a moderate response rate; however, patients with endometrioid histology and CTNNB1 mutations had more robust responses as compared to other patients ^88^. The response to everolimus in patients harboring a specific mutation was the first reported for mTOR inhibitors ^88^. Similar to other mTOR inhibitors, multiple phase II trials have incorporated temsirolimus with discouraging results. Temsirolimus has been used alone ^89^, with bevacizumab ^60^, with hormonal treatments ^90^, or in combination with chemotherapy (NCT00977574). Excess toxicity was reported in all of these studies with minimal activity.

Aside from pure mTOR inhibition, other drugs attempt to inhibit key components to the PI3K-PTEN-AKT-mTOR pathway. Pilaralisib, an orally available selective and reversible PI3K inhibitor, showed only minimal activity in a phase II study ^91^. GDC-0980, a dual PI3K/mTOR inhibitor, also showed limited antitumor activity ^92^.

Metformin, an oral biguanide known for its role in the management of diabetes, has been investigated for its role in endometrial cancer. There are epidemiologic data suggesting that metformin use lowers the rate and risk of cancer deaths among diabetic patients ^93^. Metformin has been shown to inhibit cellular proliferation and induce apoptosis, effects that are potentially through mTOR inhibition ^94– 96^. The therapeutic role of metformin is still being investigated in different disease settings, including in the neoadjuvant setting (NCT01877564), in combination with standard chemotherapy (NCT02065687), and with hormonal and mTOR agents (NCT01797523).

### Immunotherapy

As our understanding of cancer biology has evolved, focus has started to shift from the tumor itself to the microenvironment with emphasis on the concept of tumor immunogenicity. One of the most promising avenues to date is that of immune checkpoint inhibitors. Programmed cell death protein-1 (PD-1) was one of the first immune checkpoint receptors to be targeted and has been found to have very high expression in endometrial cancer. To date, there are limited data regarding the use of these agents in the treatment of endometrial cancer; however, trials are ongoing ^97^.

## Current trials

The landscape of our understanding of endometrial cancer continues to change. As such, the NRG Oncology has ongoing trials and there are continued trials from the Gynecology Oncology Group (GOG) (see Table 2).

**Table 2.** Current ongoing NRG and Gynecologic Oncology Group (GOG) trials for endometrial cancer.

Clinical Trial Identifier	Trial Title	Phase	Regimen
NCT02728258	Copanlisib in Treating Patients With Persistent or Recurrent Endometrial Cancer	II	Copanlisib, phosphoinositide 3-kinase inhibitor
NCT00942357	Carboplatin and Paclitaxel With or Without Cisplatin and Radiation Therapy in Treating Patients With Stage I, Stage II, Stage III, or Stage IVA Endometrial Cancer	III	- Cisplatin and radiation therapy with adjuvant carboplatin and paclitaxel - Carboplatin and paclitaxel
NCT00492778	Radiation Therapy With or Without Cisplatin in Treating Patients With Recurrent Endometrial Cancer	II	- Radiation therapy with brachytherapy - Cisplatin, radiation therapy with brachytherapy
NCT02065687	Paclitaxel and Carboplatin With or Without Metformin Hydrochloride in Treating Patients With Stage III, IV, or Recurrent Endometrial Cancer	II/III	- Carboplatin, paclitaxel, and metformin - Carboplatin, paclitaxel, and placebo

## Conclusion

Endometrial cancer remains the most common gynecologic malignancy in the United States. Improving our knowledge of the genetic aberrations and molecular derangements of this heterogeneous disease will allow for novel therapeutic options to be identified. Furthermore, improved surgical techniques allow for reducing morbidity associated with surgical intervention. The goal of treatment for this disease remains to maximize survival outcomes while minimizing all treatment-related morbidities; the rapid advancements in our knowledge gap will continue to allow us to achieve this goal. Moreover, focusing on the preventative measures available for endometrial cancer – like attempting to decrease the epidemic of obesity – may have larger implications on combating this disease.
